# Estimation of socioeconomic attributes from location information

**DOI:** 10.1007/s42001-020-00073-w

**Published:** 2020-06-04

**Authors:** Shohei Doi, Takayuki Mizuno, Naoya Fujiwara

**Affiliations:** 1 Waseda University, Tokyo, Japan; 2 National Institute of Informatics, Tokyo, Japan; 3 Tohoku University, Sendai, Japan; 4 The University of Tokyo, Tokyo, Japan

**Keywords:** Human behavior, Socioeconomic attributes, Location information, Machine learning, Survey data

## Abstract

Timely estimation of the distribution of socioeconomic attributes and their movement is crucial for academic as well as administrative and marketing purposes. In this study, assuming personal attributes affect human behavior and movement, we predict these attributes from location information. First, we predict the socioeconomic characteristics of individuals by supervised learning methods, i.e., logistic Lasso regression, Gaussian Naive Bayes, random forest, XGBoost, LightGBM, and support vector machine, using survey data we collected of personal attributes and frequency of visits to specific facilities, to test our conjecture. We find that gender, a crucial attribute, is as highly predictable from locations as from other sources such as social networking services, as done by existing studies. Second, we apply the model trained with the survey data to actual GPS log data to check the performance of our approach in a real-world setting. Though our approach does not perform as well as for the survey data, the results suggest that we can infer gender from a GPS log.

## Introduction

Recent technological developments in portable devices such as smartphones and car navigation systems enable us to use people’s location information for academic, administrative and marketing purposes [12, 14]. For example, border-control agencies of European countries use this kind of information to control immigrants and refugees; Germany and Denmark amended domestic laws to authorize their agencies to extract data from the cellphones of asylum seekers, and similar bills were proposed in Belgium and Austria. Also, a few years ago, the United Kingdom and Norway investigated the portable devices of refugees. Information on the movement of refugees helps us understand how integrated they are into local society and plan effective policies for them, though the intention of these governments may differ from this.

As such, information on the distribution of personal socioeconomic attributes like gender, age, and education in a specific area is necessary for administrators to make suitable policies for their areas and for companies to determine the location of new stores or products. However, because of privacy security regulations, such as the General Data Protection Regulation (GDPR) enforced by the European Union (EU), broadly available location information of smart-phones is anonymized and not associated with user attributes. Consequently, except for companies that own such raw data, it is difficult to ascertain the distribution of personal attributes.

We assume, however, that because our attributes drive our behavior and, therefore, define our location, we can reverse engineer this process. Several studies aim at stochastically predicting personal attributes from location information. For example, Lamanna et al. estimated the number of Twitter users tweeting in foreign languages in several areas, combining the residential areas of Twitter users inferred from geo-tagged tweets actively posted at night and the language of the tweets [19]. Similarly, Lenormand et al. predicted the workplaces of Twitter users from the places where they tweet during the day [21]. We can infer some personal attributes from these estimated workplaces and residences.

Instead of predicting personal attributes, some studies aim at estimating the spatial distribution of personal attributes. A most notable example of this predicts economic situations from mobile phone data [4], while others use restaurant data [9]. Beyond location information, many studies use other resources to predict individual attributes. These resources include social networking services (SNS) [3, 6, 17, 23, 26, 27], especially Twitter and Facebook, which have high-resolution and easily accessible information on personal attributes, photos [22], and mobile phone behavior [2]. In this context, our study extends these analyses by adding another source, i.e., location information, to predict personal attributes.

Drawing on these studies, we developed classifiers estimating the socioeconomic attributes of people directly from their location information. Because some studies mentioned above use SNS users, we collected a sample of Japanese citizens who reflect features of the population through a research company. Our sample includes 3000 respondents in Tokyo, which is extensive data systematically containing personal and location information. Using this sample, we trained various supervised machine learning models, including logistic Lasso regression, Gaussian Naive Bayes, random forest, XGBoost, LightGBM, and support vector machine (SVM), and compared their performance. We found it is possible to predict several attributes, including gender, from locations and XGBoost generally performed well over other methods. Moreover, we used another sample consisting of about 1000 individuals outside of Tokyo and actual GPS logs of about 150 persons to check the performance of the models for out-sample prediction.

Despite the advantage of understanding personal movement and attributes, we need to take care of the concern about privacy. It is possible that, for example, men (women) are regarded as women (men) by machine learning and this problem is more sensitive for persons with gender neutrality (LGBT). In this context, we face a trade-off: if we can perfectly predict personal attributes, we would reveal sensitive information, like sexual orientation, while if we poorly predict them, some persons may be treated in a problematic way. Keeping this possibility in mind, our probabilistic approach allows us to balance the benefit and cost of predicting personal attributes, that is, if men (women) are predicted as women (men), it is impossible to distinguish this between misclassifying and uncovering sexual orientation.

Our predictive models developed in this study contribute to advance in social survey methods using location information obtained from portable devices. In the fight against COVID-19 pandemic, for example, the government of Israel decided to “track people suspected or confirmed to have been infected with the coronavirus by monitoring their mobile phones” [13] and Baidu provide tracing data of mobile phones to understand how and why the outbreak happened [28]. In the academic field, the relation between human mobility and infection has been intensively studied not only for the novel corona virus [8, 11, 16, 18] but also others like SARS and H1N1 influenza [1, 5, 10]. Though data on human trajectory itself are useful to analyze and forecast pandemic, location information with personal socioeconomic attributes must enrich the understanding of pandemic. It is argued that elderly people tend to severely suffer from this novel coronavirus but the young are less likely to show disease and more likely to transmit it by moving around. Therefore, though it is beyond the scope of this paper, if we detect the mobility of young and elderly persons almost in a timely manner, we can find suspicious routes of infection and clusters of those who vulnerable to the virus and promptly take necessary measures. For another example, because GPS is two-dimensional information, it is hard to detect a shop or restaurant which a person visited inside a building. If we can use personal attributes estimated from other visit information, it may be possible to find a facility which he/she was most likely to visit.

The remainder of this paper proceeds as follows: in Sect. 2, we describe the two datasets used in this study: our survey data and actual GPS log data collected by other researchers. In Sect. 3, we explain the supervised learning methods to predict personal attributes and metrics for the evaluation of the performance of each method. In Sect. 4, we report the results of our analysis, particularly on gender and age, which have been intensively studied as essential attributes. We tested not only in-sample and out-sample performance using our survey data, but also applied our learner to the GPS log data.

## Data

In this study, we used two types of data: survey data and GPS log data. We collected our sample through the Rakuten Insight, Inc. research company to ensure that our sample reflects the features of the Japanese population. Because this company has a pool of respondents, their demographic information (gender, age, and residence) is registered. Our sample consisted of 3000 people in Tokyo, 400 in Miyagi, 400 in Hiroshima, and 160 in Nagasaki. In addition to Tokyo, the capital of Japan, we selected Miyagi and Hiroshima as regional central cities in the Tohoku and Chugoku areas, and Nagasaki in the Kyushu area as typical suburban cities. In 2019, Hiroshima, Miyagi, and Nagasaki were the 12th, 14th, and 30th largest prefectures out of 47 in terms of population. From the viewpoint of the means of transportation, Nationwide Person Trip Survey by Ministry of Land, Infrastructure and Transport in 2015 shows 44.2% of people in 23 wards of Tokyo move by train whereas 44.6% in Hiroshima city (the prefectural capital of Hiroshima) and 53.4% in Sendai (the prefectural capital of Miyagi) move by car (these data do not cover Nagasaki). We include these regions as well as Tokyo in the sample to ensure our data contain respondents with various features.

**Fig. 1:**
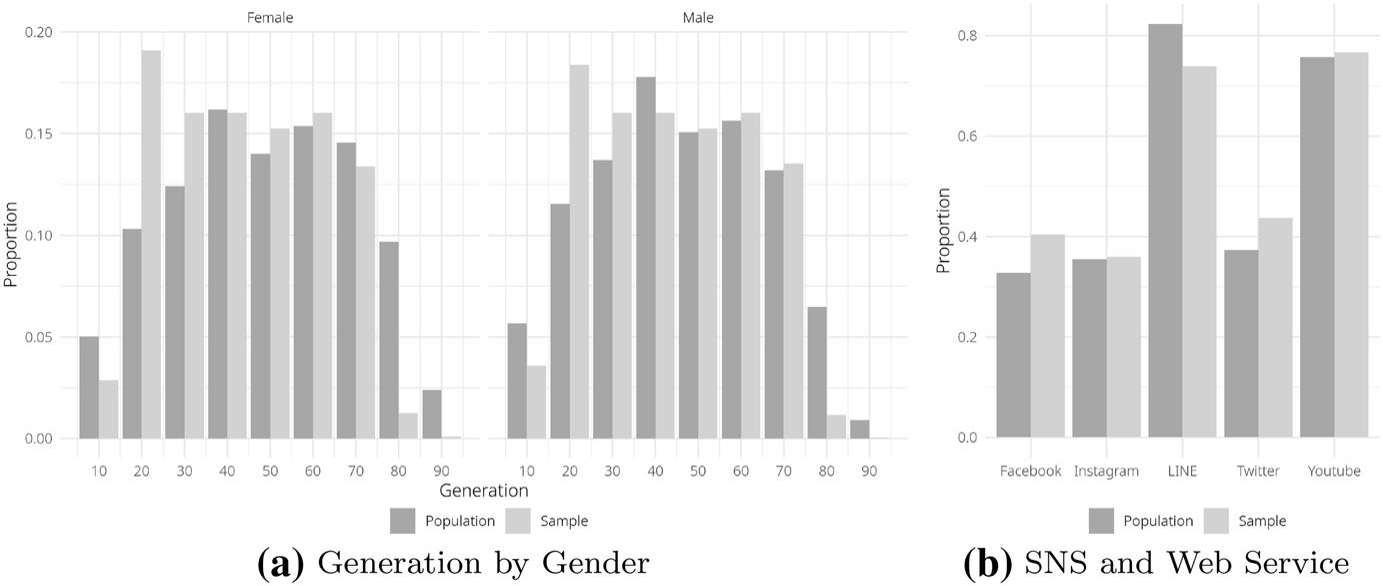
Comparison between population and sample

Figure 1a shows the proportion of each generation of Japanese males and females in the population (dark gray bars) and our sample (light gray bars). Although our sample reflects the demographic features of the population, there are more young people in their 20s and 30s and less older people in their 80s. We intentionally collected young respondents more than in the population to obtain information on the young with various backgrounds because these the 20s include students and workers and the 30s contain single and married persons, while we could not find a sufficient proportion of older people.

Because this divergence of generations may bias our sample toward the younger generation and the company collected the respondents via the internet, we also checked the usage of SNS and other internet services (Fig. 1b). We obtained the information for the population in 2019 from a report issued by the Institute for Information and Communications Policy, Ministry of Internal Affairs and Communications in Japan. Overall, the respondents in our sample use SNS and other web services as often as the entire Japanese population, which suggests that the respondents do not consist of those who more heavily use the internet than the population.

We asked each respondent 50 questions on their socioeconomic attributes as targets and 50 questions on the frequency of their visiting a specific place or facility as predictors (see the appendix for the lists of full attributes and location information). Socioeconomic attributes include gender, age, education, job, marriage status, religion, family structure, income, savings, and assets. We obtained the location information by asking the respondents how often they visit “XXX (the name of a specific facility)”. Their choices were (1) rarely, (2) once a year or less, (3) twice a year, (4) once every few months, (5) once a month, (6) a few times a month, (7) once a week, (8) a few times a week, and (9) almost every day. Additionally, because we also questioned the respondents about their residence, we collected economic and demographic information about the district they live in to use as predictors.

**Fig. 2:**
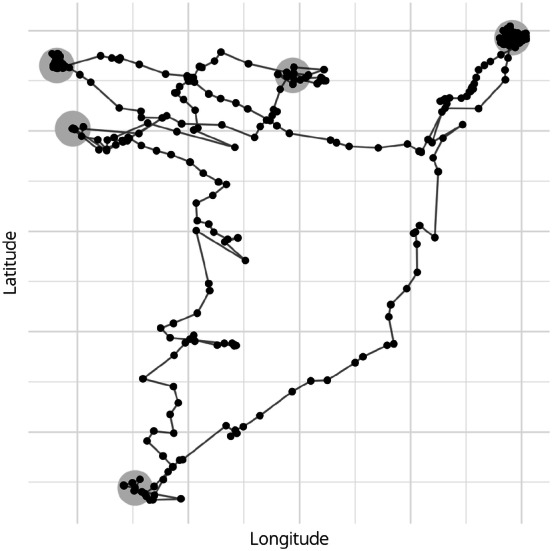
Example of stop detection from GPS log

After developing classifiers to predict socioeconomic attributes from location information using this survey data, we applied these predictive models to actual GPS log data [15]. They gathered the latitude and longitude of 184 respondents in the Kanto region (including Tokyo) almost every 5 min from the respondents’ mobile phones from November 28 to December 22, 2011, estimated the rectangle of stay information (i.e., the maximum and minimum latitude and longitude), and asked the respondents to report the name of the estimated locations voluntarily. Because our purpose is to estimate personal attributes from the GPS log itself, instead of using this stay information, we assume a respondent stayed if they moved only within a radius of 100 m for at least 20 min [24]. Figure 2 shows an example of an actual GPS log (black dots) and detected stops (grey circles) of one of the authors (not obtained from the dataset). The dataset also contains the names and the rectangles (i.e., the maximum and minimum latitude and longitude) of many facilities the respondents visited. By coding the category of the facilities’ names to match those in our survey data, we made the correspondence table between the rectangle and category of a facility. Based on this table, we checked if the latitude and longitude of the estimated stay from the GPS logs were located in the rectangle of a facility, and calculated the frequency of visiting each category of facilities.

## Methods

We used several supervised learning methods and compared their performance: logistic Lasso regression, Gaussian Naive Bayes, random forest, XGBoost, LightGBM, and support vector machine (SVM) with a radial basis function. Because we have about 50 targets to predict, we use several common hyperparameters of these models and the default settings in scikit-learn [25] and imbalanced-learn [20], not tune them for each target. Therefore, the performance we show in the next section could be improved by tuning the hyperparameters more precisely for each target.

We briefly describe each method we use in this study as follows. Let $y_i$ and $X_i = (x_{i1},\ldots ,x_{im})$ denote a target (i.e., an attribute) and a vector of features (i.e., visiting and district information) for person *i*. For visiting information, $x_{ij} \in \{0,\ldots ,8\}$ is the response to the questions on the frequency of person *i* visiting facility *j*, where 1 implies he/she rarely visits there and 9 implies he/she visits there almost every day. Other components of an input vector are economic, social, demographic, and geographical district-level information of the residence of the respondents. The full list of target variables and input features appears in Appendix.

For simplicity, we assume that a target is binary, $y_i \in \{0,1\}$, in this section, but the methods can be applied to a multinomial target. In logistic regression, the conditional probability is given as a logistic function,

1 $$\begin{aligned} \mathrm {Pr}(y_i=1 \mid X_i) = \frac{e^{X_i\beta }}{1+e^{X_i\beta }} = \frac{1}{1+e^{-X_i\beta }}, \end{aligned}$$

where $\beta$ is a vector of coefficients for the features. Then, we obtain the cross-entropy to minimize as

2 $$\begin{aligned} -\sum _i \left\{ y_i \log \mathrm {Pr}(y_i=1 \mid X_i) + (1-y_i) \log \mathrm {Pr}(y_i=0 \mid X_i)\right\} . \end{aligned}$$

In logistic Lasso regression, we add a penalty term, $\lambda |\beta |$, to this loss function. Intuitively, because the loss function increases as the coefficients become large, this penalty term makes the coefficients “shrink” more and the regularization parameter, $\lambda$, determines the degree of shrinkage. In the following analysis, the regularization strength, $\lambda$, in the objective function is 0.1, 1, 10, or 100.

Gaussian Naive Bayes is a simple classification method based on the Bayes theorem. According to this theorem, we obtain the conditional probability as

3 $$\begin{aligned} \mathrm {Pr}(y_i \mid X_i) = \frac{\mathrm {Pr}(X_i \mid y_i)\mathrm {Pr}(y_i)}{\mathrm {Pr}(X_i)} \end{aligned}$$

and, if elements of $X_i$ are independently and normally distributed, the likelihood in the numerator becomes

4 $$\begin{aligned} \mathrm {Pr}(X_i \mid y_i) = \prod _j \phi \left( x_{ij} \mid \mu _y, \sigma _y^2\right) , \end{aligned}$$

where $\phi (\cdot )$ is the probability density function of Gaussian distribution, and $\mu _y$ and $\sigma _y^2$ are the mean and variance of this feature in class *y*. Because we can simply estimate these parameters from data by the maximum likelihood, we have no hyperparameters to tune with this method.

Random forest, XGBoost, and LightGBM are ensemble learning based on a decision tree, which is a predictive method to find partitions corresponding to the class of a target according to the value of the features. Random forest, XGBoost, and LightGBM construct multiple weak decision trees and predict a target by the mode of the classes predicted by them. While random forest parallelly creates weak learners, XGBoost and LightGBM employ gradient boosting, which sequentially generates weak learners using the result of the previous one. For random forest, we have a variety of hyperparameters, but only choose the number of trees from 10, 100, and 1000, whereas we do not tune any parameters for XGBoost and LightGBM.

SVM is a supervised learning method to obtain a hyperplane that linearly separates feature space into positive and negative cases. If the sets are not linearly separable, we can construct a non-linear classifier using a kernel trick and the Gaussian (radial basis function) kernel, $\exp (-\gamma |X_i-X_j|^2)$, is a well-known kernel function. In the following analysis, the regularization parameter (as discussed in Lasso), $\lambda$, is 50 or 100 and the kernel coefficient, $\gamma$, in the Gaussian kernel function is also 0.01 or 0.02.

Some targets in our survey data are imbalanced in that most cases are negative, while only a few are positive. For example, only 2.2% of the respondents in our data answered that practicing martial arts is their hobby. If we predicted that nobody likes to practice martial arts, we got 97.8% accuracy, but this result is misleading or meaningless. To deal with this problem of imbalanced data, we used the synthetic minority over-sampling technique (SMOTE), which increases positive (or minority) cases by interpolating [7]. For one minority case, SMOTE randomly draws one case from its *k*-neighbors and creates an artificial data point between an initial one and a selected one. Repeating this process, SMOTE increases the number of minority cases up to that of the majority.

**Table 1:** Confusion matrix

		Ground truth	
		$y_i = 1$	$y_i = 0$
Prediction	${\hat{y}}_i = 1$	True positive (TP)	False positive (FP)
	${\hat{y}}_i = 0$	False negative (FN)	True negative (TN)

Moreover, we rely on not only accuracy, but also other metrics considered to be more robust to imbalance, like the *F* score, the area under the receiver operating characteristic curve (ROC AUC) and precision–recall curve (PR AUC), and the Matthews correlation coefficient (MCC). In the confusion matrix (Table 1), we have four strata according to the ground truth, $y_i$, and the prediction, ${\hat{y}}_i$: true positive (TP), true negative (TN), false positive (FP), and false negative (FN). Accuracy is the ratio of correctly predicted cases:

5 $$\begin{aligned} \text {Accuracy} = \frac{\text {TP} + \text {TN}}{\text {TP} + \text {TN} + \text {FP} + \text {FN}}. \end{aligned}$$

*F* score is the harmonic mean of precision and recall, where

6 $$\begin{aligned} \text {Precision}&= \frac{\text {TP}}{\text {TP} + \text {FP}} \end{aligned}$$

7 $$\begin{aligned} \text {Recall}&= \frac{\text {TP}}{\text {TP} + \text {FN}}. \end{aligned}$$

MCC is also known as the $\phi$ coefficient of the $2 \times 2$ contingency matrix, which we can obtain by

8 $$\begin{aligned} \text {MCC} = \frac{\text {TP}\cdot \text {TN}-\text {FP}\cdot \text {FN}}{\sqrt{(\text {TP} +\text {FP})(\text {TP}+\text {FN})(\text {TN}+\text {FP})(\text {TN}+\text {FN})}}. \end{aligned}$$

Before defining ROC AUC and PR AUC, we introduce the false-positive rate, $r_{\text {fp}}$, and true-positive rate, $r_{\text {tp}}$,

9 $$\begin{aligned} r_{\text {fp}}&= \frac{\text {FP}}{\text {TN} + \text {FP}} \end{aligned}$$

10 $$\begin{aligned} r_{\text {tp}}&= \frac{\text {TP}}{\text {TP} + \text {FN}}, \end{aligned}$$

and suppose that there exists some threshold, *p*, such that if the predicted probability of an individual being positive, ${\hat{p}}_i$, is greater than *p*, we predict that the individual is positive. Then the true-positive and false-positive rates depend on this threshold and we obtain the ROC curve, $(r_{\text {fp}}(p), r_{\text {tp}}(p))$ and area under the ROC curve. Similarly, we obtain the area under the PR curve, $(\text {Precision}(p), \text {Recall}(p))$. For multiclass targets, e.g., job, we do not calculate the *F* score, ROC AUC, and PR AUC, and for the continuous target, only age in this study, we use the root mean squared error (RMSE), $\sqrt{\frac{1}{N}\sum _i (y_i-{\hat{y}}_i)^2}$, and mean absolute error (MAE), $\frac{1}{N}\sum _i |y_i-{\hat{y}}_i|$, as metrics.

We evaluated the performance in several ways. We conducted fivefold cross-validation for the Tokyo sample and averaged those metrics to check the in-sample performance. Then we trained the predictive models using the whole Tokyo sample and tested the out-sample performance with samples from the other three regions. Finally, we checked the performance in a real-world setting with the GPS log data. Note that we oversampled only the training set, not the test set, and standardized and scaled both the training and test sets so that all features had a mean of one and a variance of zero.

**Fig. 3:**
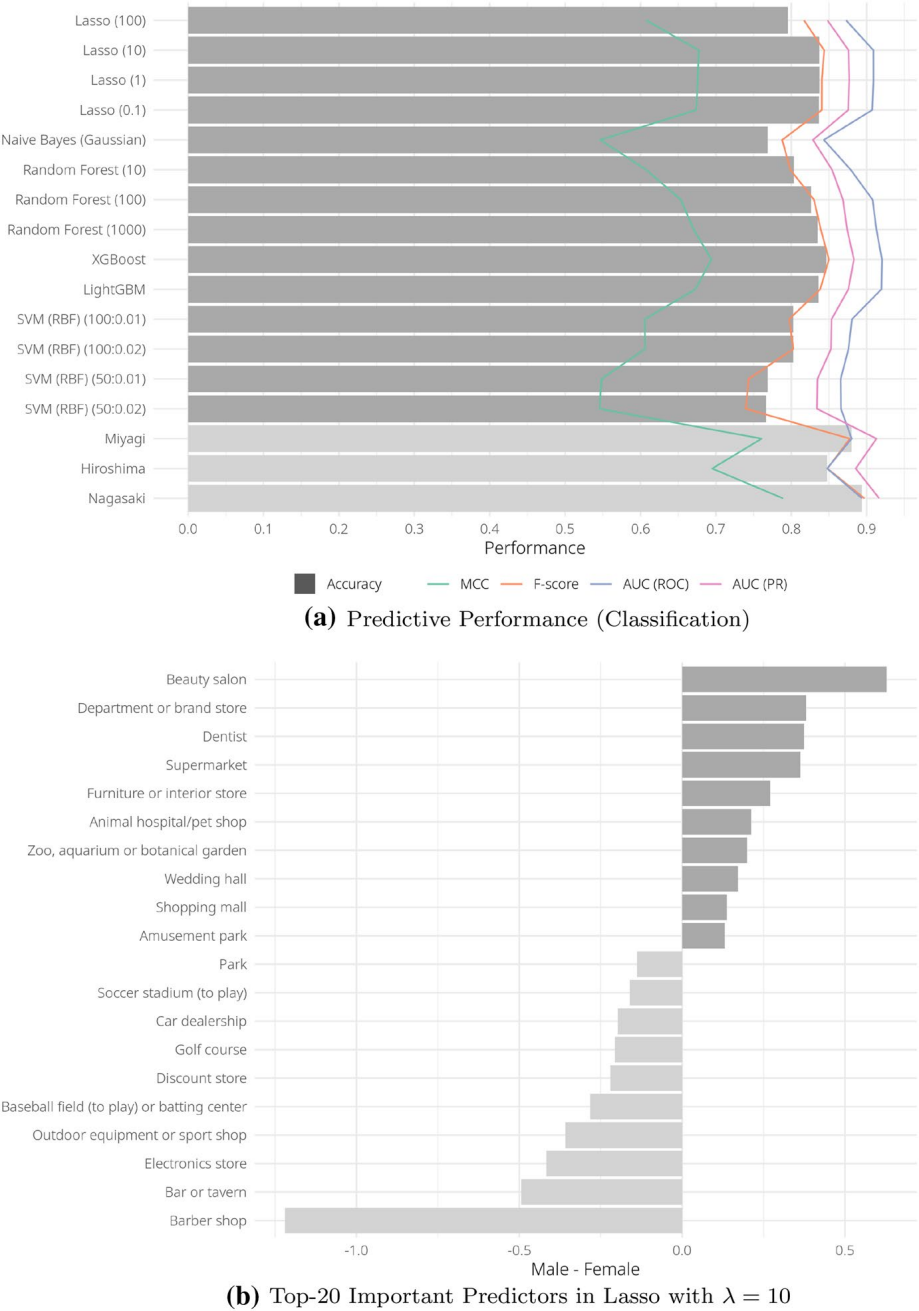
Prediction of gender

## Results

We investigated the gender and age predictions in detail because these are fundamental attributes that the existing studies mentioned above also tried to predict. Figure 3a shows the results of predicting gender from location information; the dark gray bars are the accuracy of each method for the Tokyo sample and the light gray bars are those for the samples outside Tokyo by XGBoost. Looking at the accuracy (because gender is balanced in our sample), the classifiers predicted gender with about 80% accuracy on average and XGBoost shows the highest performance with accuracy of 0.8463, *F* score of 0.8498, and ROC AUC of 0.9202, which is as accurate as the existing studies. For example, the ROC AUC of Koniski et al. using “Facebook Likes”, one of the prominent studies on SNS and personal attributes, is 0.93 [17]. In general, the average accuracy and *F* score of the gender prediction from the SNS information were 0.83 and 0.84, according to the survey article [6]. Moreover, the out-sample accuracy for three prefectures is higher than the in-sample one, probably because we used the entire Tokyo sample to train for these cases and there is no “metropolitan” bias.

To see how strongly and in which direction location information is associated with gender, we further investigated the coefficients of predictors in the logistic Lasso regression when the regularization parameter is 10 (Fig. 3b). We used the Lasso results because, unlike tree-based methods, Lasso shows not only the strength of the predictors, but also the sign of the coefficients. In the figure, the dark gray bars show the coefficient of features related to females and the light gray ones are those for males. The most significant but trivial factor was whether to go to a barbershop or beauty salon. More interestingly, we can predict gender from buying behavior; that is, those who frequently go to department stores, furniture stores, or supermarkets are likely to be regarded as female, while those who go to electronic, sport, or discount stores are considered male.

**Fig. 4:**
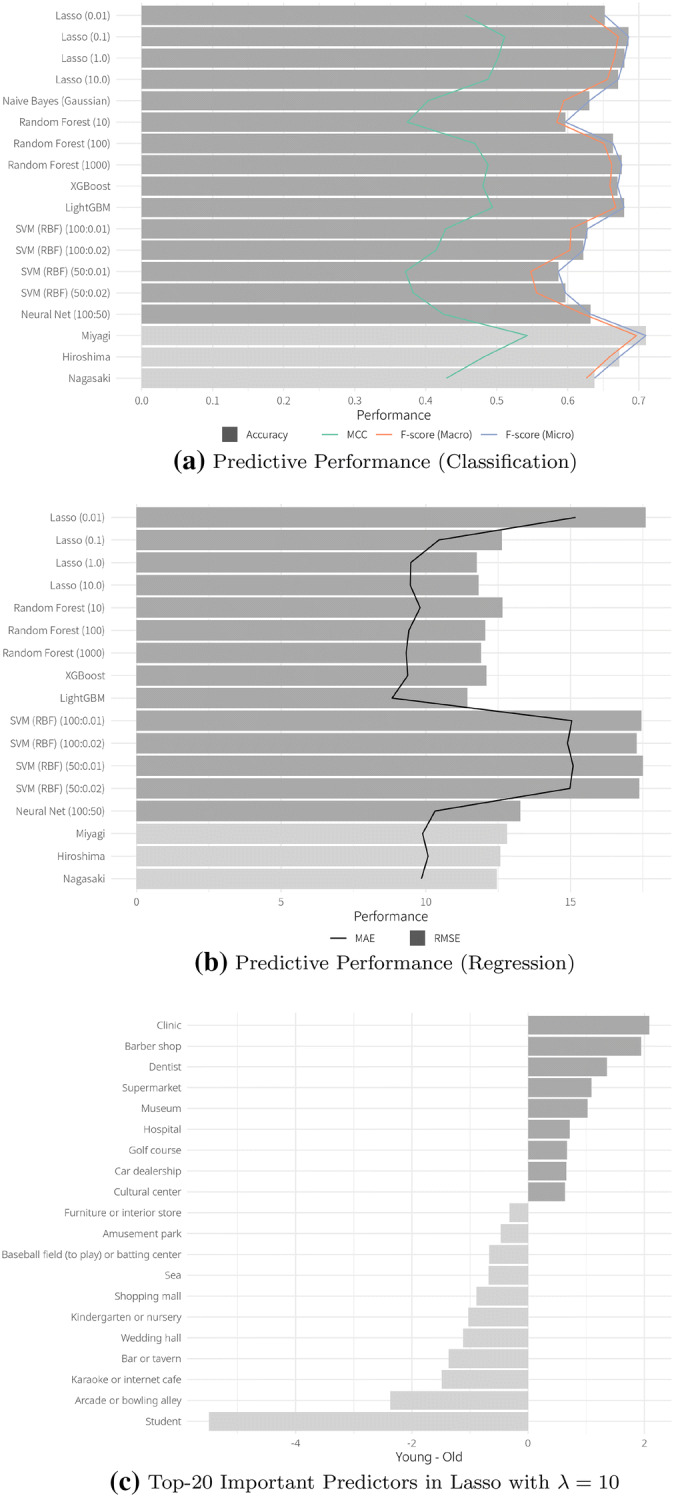
Prediction of age

Figure 4a, b shows the result of the generation and age prediction. First, we split the sample into three groups—young (under 29), middle-aged (between 30 and 59), and elderly (over 60) persons—because, as we mentioned in “Introduction”, tracing routes of and detecting cluster of young and elderly people is crucial in dealing with the coronavirus pandemic. The accuracy and MCC for several models to predict generation are almost 0.68% and 0.5 but this result is less intuitive because the generation is multinomial variable. Second, therefore, we predict it as a continuous variable by regression and use RMSE and MAE as metrics to evaluate the performance of each method. MAEs are no less than about nine, which implies that location information hardly predicts 10-year age groups but, combining the first result, it is still useful in predicting boarder generation. Again, there is no systematic difference between in-sample and out-sample predictions for both predictions. In Fig. 4c, the dark gray and light gray bars represent the coefficient of the predictors associated with older and young people, respectively. Other than whether they are a student, facilities related to hobby seem strongly correlated with young people, e.g., arcade, bowling alley, karaoke, internet cafe, and bar. In contrast, older people tend to go to facilities related to health, e.g., clinic, dentist, and hospital. Though the accuracy leaves room for improvement, the result is intuitive and suggests that location information could provide us with an individual’s age.

**Fig. 5:**
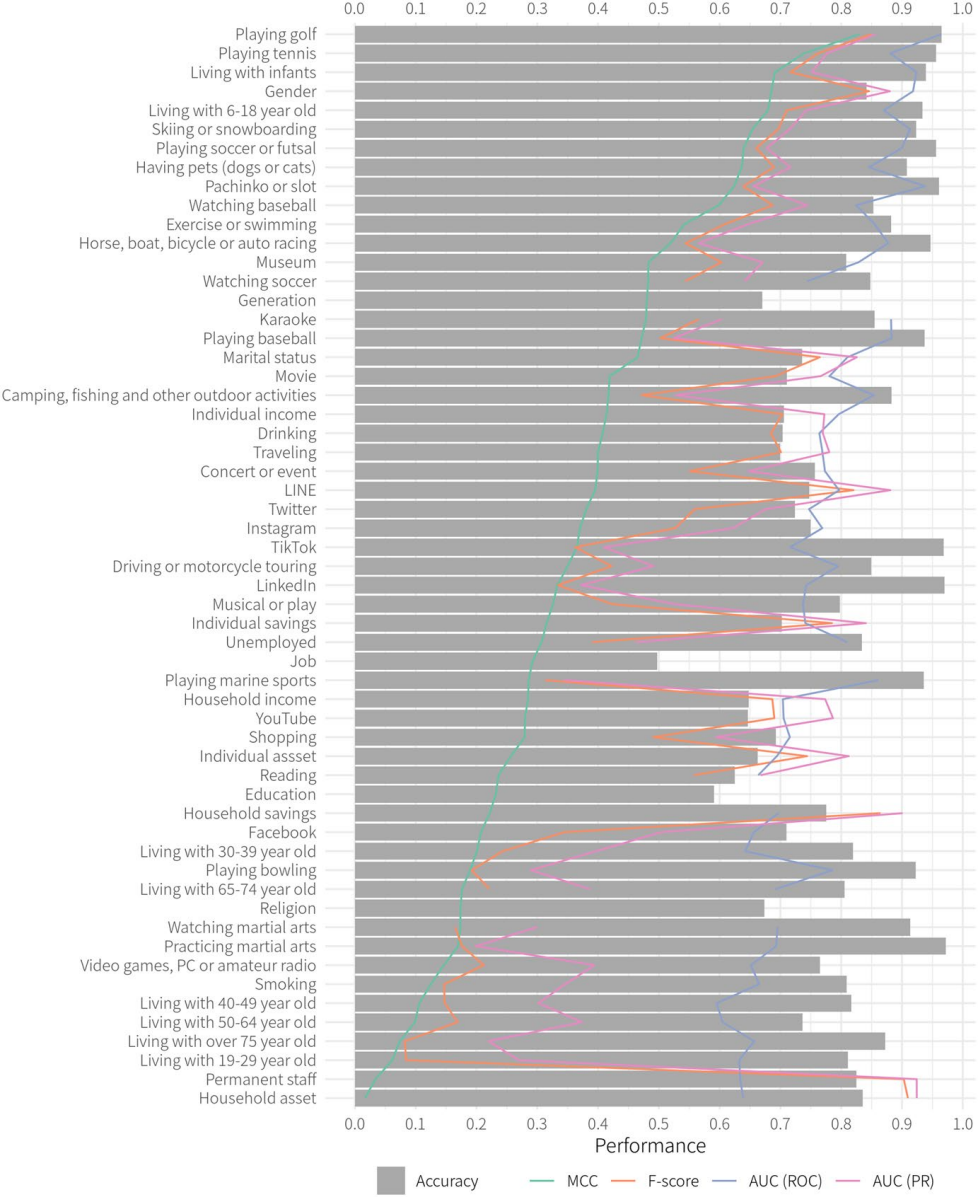
Overall in-sample performance of XGBoost for all attributes

We compared the overall in-sample performance for all targets by XGBoost because this method performed reasonably well on average. XGBoost shows higher performance in terms of MCC probably for two reasons. First, generally speaking, tree-based approaches, i.e., Random Forest, XGBoost, and LightGBM, are less sensitive to the scale of the value of input features because they recursively split a sample into several groups with a threshold during train. In contrast, logistic (Lasso) regression assumes the same interval of value implies the same weight. For example, the frequency of visit is coded as an integer from 1 to 9 in our dataset but the difference between 1 (rarely) and 2 (once a year or less) and that between 8 (a few times a week) and 9 (almost every day) may not have substantively equal importance for predicting personal attributes. Second, XGBoost and LightGBM employ gradient boosting, which generates a weak learner reflecting errors by previous weak learners during train while Random Forest uses bagging, which parallelly generates weak learners. As a result, we consider XGBoost shows high performance (though LightBGM also has slightly lower predictive power).

In Fig. 5, the targets are arranged in order of the value of MCC from top to bottom. Gender, living with infants and children, hobbies that require specific facilities (e.g., playing golf or tennis), and gambling (pachinko and race) are highly predictable. Although it is difficult to set criteria for the strength of the correlation coefficient, we may predict other important socioeconomic attributes like marital status and individual income and savings. In contrast, living with adults or older people and hobbies not related to a specific place (e.g., playing video games and smoking) are difficult to predict from location information only.

**Table 2:** Comparison between survey data and GPS log

*(a) Prediction of gender*		
Metrics	Survey	GPS
Accuracy	0.735333	0.629371
MCC	0.472466	0.291346
AUC (ROC)	0.824475	0.637745
AUC (PR)	0.803007	0.737777
*F* score	0.746033	0.569106
*(b) Prediction of generation*		
Accuracy	0.747094	0.474820
MCC	0.404128	0.109331
AUC (ROC)	0.772475	0.556818
AUC (PR)	0.875023	0.799741
*F* score	0.818239	0.496552

Finally, we predicted gender and generation from the GPS log, applying XGBoost trained by our survey data from Tokyo, and compared it with the cross-validation prediction. Note that, because the GPS log data coverage is 1 month and does not contain information on residence, we trained the learner again after replacing the answers of visiting a specific facility less than once a month with those of never visiting in the survey data and dropping the district-level variables (therefore, the performance for the survey data differs from that in the previous figures). In addition, when it comes to predicting generation, the GPS log data contain only young and middle-aged persons, so that we drop the sample of elderly people from the training set. Although the prediction of gender by the GPS log is less accurate than by the survey data, gender is still a predictable attribute from a location history with accuracy of 0.63% (Table 2a). In contrast, GPS log can predict generation less accurately than survey data (Table 2b). The performance with GPS data is not as high as that with the survey data, probably because (1) the GPS log data only covers 1 month and (2) the respondents in the GPS data went to facilities we did not ask in the survey, both of which can be solved by improving the data collection procedure. Moreover, if we incorporate the sequence of locations into classifiers, the performance must be improved.

## Conclusion

In this study, we tested our conjecture that locations hint at personal attributes. To this end, we collected a comprehensive dataset of personal attributes and location information in Japan and showed that it is possible to estimate socioeconomic attributes, including gender, living with infants and children, marital status, and individual income, from the frequency of visits to facilities. We also applied our predictive model to location information we extracted from real GPS log data to check the performance in a realistic situation and found that the performance of predicting gender is predictable to some extent, though less than from the survey data. Overall, our analysis suggests that socioeconomic attributes affect human behavior and, therefore, human location.

At the same time, our study poses several limitations. First, we need more extended time coverage of the GPS log to predict attributes as accurately as from the survey data. Second, we found that the respondents in the GPS data visited places or facilities that we did not ask about while collecting survey data, but which might have information on their characteristics. Third, if we use the sequence of human movement in prediction, the performance must be higher than our result in this paper, but trajectory data with personal attributes are hard to access for researchers. All require a more elaborate data-collecting process in the training and test sets. Nevertheless, our study shows how accurately socioeconomic attributes can be predicted just by the frequency of visiting facilities and places. Because more and more companies collect information on the movement of customers from portable devices, if these companies provide researchers with this information as Twitter does for SNS, the analysis of human movement will be a major topic in computational social science.

Finally, we believe that our study has the potential for policy implications in political, economic, and social contexts. Moreover, as we discussed in “Introduction”, location information has been used in infection prevention and immigration control. For infection prevention of COVID-19, understanding what kind of people are going where is important in forecasting and preventing a pandemic. Presumably, the movement of young people is suspected of routes of transmission and places where elderly people gather can be at higher risk of infection. For refugee and immigration policy, by applying our approach to citizens, we can understand what kind of people or communities interact with which part of immigrant society. This sort of analysis, we believe, helps policymakers integrate immigrants into a host country, relaxing disputes among them, and improving social welfare. We need to seek a way of balancing between the protection of privacy and the utility of information for a better society.

## Appendix: List of variables

See Tables 3, 4 and 5.

**Table 3:** List of location information

Facility	
Laundry	
Supermarket	
Electronics store	
Outdoor equipment or sport shop	
Furniture or interior store	
DIY store	
Discount store	
Department or brand store	
Shopping mall	
Car dealership	
Kindergarten or nursery	
Elementary, middle or high school	
University or college	
Cultural center	
Language school	
Dentist	
Clinic	
Hospital	
Nursing care store	
Animal hospital/pet shop	
Baseball field (to play) or batting center	
Soccer stadium (to play)	
Baseball field (to watch)	
Soccer stadium (to watch)	
Event hall or theater	
Golf course	
Tennis court	
Pool or gym	
Executive hotel	
Hot spring	
Camp site	
Ski area	
Sea	
Zoo, aquarium or botanical garden	
Museum	
Park	
Amusement park	
Arcade or bowling alley	
Racecourse	
Pachinko or slots	
Movie theater	
Bar or tavern	
Beauty salon	
Barber shop	
Restaurant	
Karaoke or internet cafe	
Shrine or temple	
Church	
Wedding hall	
Funeral hall

**Table 4:** List of district information

Category	
Average income	
Distance from City Hall to Tokyo Station	
Travel time from City Hall to Tokyo Station	
Fare from City Hall to Tokyo Station	
Average land price	
Crime rate	
Average household size	
Foreign people ratio	
Population	
Population (0–9 years old)	
Population (10–19 years old)	
Population (20–29 years old)	
Population (30–39 years old)	
Population (40–49 years old)	
Population (50–59 years old)	
Population (60–69 years old)	
Population (70–79 years old)	
Population (80–89 years old)	
Population (over 90 years old)

**Table 5:** List of socioeconomic attributes

Attribute	Note
Gender	Female or male
Age	
Marital status	Single or married
Job	Employee, civil servant, self-employed, part-time worker, housewife or other
Unemployed	Unemployed or not
Permanent staff	Permanent or not
Education	Undergraduate, graduate or other
Religion	Buddhist, Christian, other or no religion
Drinking	More than once a month or less
Smoking	Every day or less
Facebook	More than once a month or less
Twitter	More than once a month or less
Instagram	More than once a month or less
YouTube	More than once a month or less
LINE	More than once a month or less
LinkedIn	More than once a month or less
TikTok	More than once a month or less
Living with infants	yes or no
Living with 6–18 year old	Yes or no
Living with 19–29 year old	Yes or no
Living with 30–39 year old	Yes or no
Living with 40–49 year old	Yes or no
Living with 50–64 year old	Yes or no
Living with 65–74 year old	Yes or no
Living with over 75 years old	More than one or not
Individual income	More than 9 million yen or less
Household income	Less than 1.2 million, between 1.2 million and 2 million yen or more than 2 million yen
Individual savings	Less than 1.2 million, between 1.2 million and 2 million yen or more than 2 million yen
Household savings	Less than 1.2 million, between 1.2 million and 2 million yen or more than 2 million yen
Individual asset	Less than 1.2 million, between 1.2 million and 2 million yen or more than 2 million yen
Household asset	Less than 1.2 million, between 1.2 million and 2 million yen or more than 2 million yen
Playing baseball	Yes or no
Playing soccer or futsal	Yes or no
Practicing martial arts	Yes or no
Playing golf	Yes or no
Playing bowling	Yes or no
Playing tennis	Yes or no
Playing marine sports	Yes or no
Skiing or snowboarding	Yes or no
Camping, fishing and other outdoor activities	Yes or no
Watching baseball	Yes or no
Watching soccer	Yes or no
Watching martial arts	Yes or no
Movie	Yes or no
Museum	Yes or no
Concert or event	Yes or no
Musical or play	Yes or no
Reading	Yes or no
Video games, PC or amateur radio	Yes or no
Exercise or swimming	Yes or no
Shopping	Yes or no
Pachinko or slot	Yes or no
Horse, boat, bicycle or auto racing	Yes or no
Karaoke	Yes or no
Traveling	Yes or no
Driving or motorcycle touring	Yes or no
Having pets (dogs or cats)	Yes or no
